# Association of Population Screening for Breast Cancer Risk With Use of Mammography Among Women in Medically Underserved Racial and Ethnic Minority Groups

**DOI:** 10.1001/jamanetworkopen.2021.23751

**Published:** 2021-09-10

**Authors:** Candice Schwartz, Ifeanyi Beverly Chukwudozie, Silvia Tejeda, Ganga Vijayasiri, Ivy Abraham, Mylene Remo, Hiral A. Shah, Maria Rojas, Alicia Carillo, Loraine Moreno, Richard B. Warnecke, Kent F. Hoskins

**Affiliations:** 1 Division of Hematology/Oncology, University of Illinois at Chicago; 2 University of Illinois Cancer Center, Chicago; 3 Institute for Health Research and Policy, University of Illinois at Chicago; 4 Division of Geriatric and Palliative Medicine, University of Michigan, Ann Arbor; 5 Now with Primary Healthcare Associates SC, Harvey, Illinois; 6 Now with Affiliated Oncologists, Tinley Park, Illinois; 7 Now with Ohio Health, Mansfield, Ohio; 8 Chicago Family Health Center, Chicago, Illinois; 9 Translational Oncology Program, University of Illinois Cancer Center, Chicago

## Abstract

**Importance:**

Black women bear a disproportionate burden of breast cancer mortality in the US, in part due to inequities in the use of mammography. Population screening for breast cancer risk in primary care is a promising strategy for mitigating breast cancer disparities, but it is unknown whether this strategy would be associated with increased mammography rates in underserved women of racial and ethnic minority groups.

**Objective:**

To examine whether providing individualized breast cancer risk estimates is associated with an increase in the rate of screening mammography.

**Design, Setting, and Participants:**

A cohort study was conducted in women receiving individualized risk estimates as part of routine primary health care at federally qualified health centers in medically underserved communities in Chicago, Illinois. The study was conducted from November 5, 2013, to December 19, 2014, with data acquisition completed on March 5, 2017; data analysis was performed from December 30, 2020, to February 2, 2021. A total of 347 women aged 25 to 69 years without a personal history of breast cancer presenting for an annual visit with their primary care clinician were enrolled.

**Exposures:**

Breast cancer risk estimates were obtained with validated risk assessment tools as a standard component of the clinic check-in process. One of 4 women at average risk and all women at high risk were invited to participate in the study.

**Main Outcomes and Measures:**

The primary outcome was the mammography rate during 18 months of usual care compared with the rate during 18 months after implementation of risk assessment.

**Results:**

Of the 347 women enrolled, 188 were age-eligible for mammography and were included in the analysis (mean [SD] age, 50.8 [7.04] years); 70 women (37.2%) were Hispanic, 114 (60.6%) were non-Hispanic African American, and 4 (2.1%) were from other racial and ethnic groups (4 non-Hispanic White women). Ninety-eight women (52.1%) had an average risk of developing breast cancer and 90 (47.9%) were at high risk. Overall, there was a nonsignificant increase in the mammography rate, from 38.8% during usual care to 48.9% following implementation of risk assessment (odds ratio, 1.37; 95% CI, 0.92-2.03). In preplanned subgroup analysis, the mammography rate among women at high risk was significantly higher after vs before risk assessment (51.1% vs 36.6%; odds ratio, 1.88; 95% CI, 1.10-3.23).

**Conclusions and Relevance:**

In this study, providing individualized breast cancer risk estimates as a component of primary health care in federally qualified health centers was associated with increased use of mammography among women of racial and ethnic minority groups who were at high risk. Implementation of this approach in underserved communities could promote equity in the use of mammography and reduce racial disparities in breast cancer mortality. This strategy warrants further investigation.

## Key Points

**Question:**

Is population screening for breast cancer risk associated with increased use of mammography among medically underserved women from racial and ethnic minority groups?

**Findings:**

In this cohort study including 188 women, providing individualized breast cancer risk estimates as a standard component of annual preventive health care was associated with improved use of mammography among women at high risk. This group’s rate of annual mammography increased from 37% during usual care to 51% following risk assessment.

**Meaning:**

The findings of this study suggest that providing individualized breast cancer risk estimates as a standard component of preventive health care may reduce racial inequities in breast cancer screening and ultimately mitigate disparities in breast cancer mortality.

## Teaser

This cohort study assesses mammography use in a sample of women from participating federally qualified health centers to examine whether providing individualized breast cancer risk estimates is associated with increased uptake of cancer control measures.

## Introduction

Widespread implementation of screening mammography contributed to the decrease in breast cancer mortality in the US over the past 30 years,^1^ but not all women benefited equally. Racial disparities in breast cancer mortality emerged as routine screening mammography became the standard of care, and more than 3 decades later the breast cancer mortality rate for Black women remains 40% higher compared with non-Hispanic White women.^2^ Racial stratification in the US produces inequities in health care and health outcomes through multilevel social determinants of health.^3^ Black women in the US historically have had lower rates of screening mammography,^4^ which leads to a higher proportion of late-stage diagnoses and lower survival rates.^5^ Data have shown a narrowing of the racial gap from 1987 to 2015, but lower screening rates persist among women of racial and ethnic minority groups with socioeconomic disadvantage.^6^

Models of health behavior change, such as the health beliefs model, have been used to identify factors associated with the uptake of mammography in women of underserved racial and ethnic minority groups.^7,8^ A key domain in the health beliefs model is perceived risk.^9^ This theoretical framework posits that a woman will be more likely to engage in breast cancer screening programs if she believes that she is susceptible to breast cancer. Earlier work reported that Black women overall were significantly less likely to perceive their breast cancer risk accurately^10^ and that Black women with low income who were nonadherent to screening recommendations were less likely to perceive themselves as susceptible to breast cancer.^11^ These data suggest that providing underserved Black women with individualized breast cancer risk estimates may promote uptake of mammography.

Cancer genetic risk assessment involves in-depth evaluation with genetic counseling and germline genetic testing of cancer susceptibility genes, and empirical risk model prediction when a pathogenic genetic variant is not identified.^12^ Women at high risk are candidates for a variety of enhanced cancer control measures.^13,14,15,16,17^ Formal cancer genetic risk assessment is a relatively time-intensive process and requires specialized expertise.^12^ However, it is possible to perform rapid screening for breast cancer risk (breast cancer risk assessment [BCRA]) in the primary care setting with validated instruments to identify women at high risk who are eligible for referral for genetic counseling and genetic testing for hereditary breast cancer syndromes based on their family history^15,18^ and are candidates for enhanced breast cancer screening with breast magnetic resonance imaging^19,20^ and pharmacologic risk reduction.^21^ Generating individualized breast cancer risk estimates for asymptomatic women in the primary care setting also provides an opportunity to ensure that the women who benefit most from mammography (ie, those with increased risk) are engaged in screening programs. To our knowledge, incorporating BCRA into preventive health care to identify high-risk individuals has not been tested as a strategy to increase mammography use among women of underserved racial and ethnic minority groups. We conducted a study to examine the feasibility of implementing BCRA as a standard component of the annual well visit at federally qualified health centers (FQHCs) in underserved racial and ethnic minority communities.^22^ Herein, we report an analysis of mammography use in a sample of women from participating FQHCs who enrolled in a prospective cohort study to examine whether providing individualized breast cancer risk estimates is associated with increased uptake of cancer control measures.

## Methods

### Study Design and Population

From November 5, 2013, through December 19, 2014, we tested the feasibility of implementing BCRA as a standard component of annual well visits and new-patient visits at 2 outpatient clinic sites of an FQHC in Chicago, Illinois.^22^ A prospective cohort study was embedded within the risk assessment feasibility study by selecting a sample of women from the entire group who received individualized risk estimates to participate in the prospective study; 1 of 4 randomly selected women at average risk and all of those at high risk were invited to participate in the prospective cohort study immediately after completion of risk assessment. Study recruiters were aware of each woman’s risk status, but participants were not aware of their individualized risk estimate at the time they were invited to participate in this study. Self-identified race and ethnicity of study participants was categorized as Asian or Pacific Islander, Hispanic, multiracial, non-Hispanic African American, non-Hispanic White, or other/do not know/refused. Study participation was restricted to women who could complete the enrollment interview in either English or Spanish. Recruitment occurred from November 5, 2013, to December 19, 2014, with data acquisition completed on March 5, 2017; data analysis was performed from December 30, 2020, to February 2, 2021. This report focuses on adherence to standard screening with mammography; therefore, the analysis reported herein includes only the subgroup of study participants who were aged 40 years or older at the time of enrollment and so were eligible for mammography according to the participating clinics’ guidelines. Younger women with increased risk were followed up for other outcomes (ie, genetic counseling referral and attendance) that were reported previously.^22^ All study participants were considered medically underserved because they receive regular care at an FQHC, and a key criterion for designation as an FQHC is that a community clinic provides primary care services in a medically underserved area.^23^

The study was approved by the institutional review board at the University of Illinois at Chicago, and all participants provided written informed consent; participants received financial compensation. This study followed the Strengthening the Reporting of Observational Studies in Epidemiology (STROBE) reporting guideline for cohort studies.^24^

### Risk Assessment, Outcomes, and Measures

Breast cancer risk assessment was performed by nonlicensed clinic staff for all women aged 25 to 69 years who did not have a personal history of breast cancer on presentation for an annual well visit or new-patient visit with their primary care clinician. Clinic staff members entered risk factor data for the assessment using tablet computers, based on patient self-report of family history and other breast cancer risk factors. Breast cancer risk assessment was performed with a custom software application developed by study investigators that collects family cancer history and nonfamilial breast cancer risk factors and integrates several validated instruments to generate a comprehensive assessment of breast cancer risk (eTable 1 in the Supplement) following input of family history and other breast cancer risk factors. The assessment tool includes the modified version of the Gail Model,^21^ the CARE model,^25^ the Claus model,^19^ the Pedigree Assessment Tool,^18^ and the National Comprehensive Cancer Network Clinical Practice Guidelines for Genetic/Familial High-Risk Assessment: Breast and Ovarian, version 1.2011.^26^ Participants were classified as high risk if the assessment indicated any of the following: family history of breast or ovarian cancer qualified them for genetic counseling referral based on the Pedigree Assessment Tool or for genetic testing for the hereditary breast and ovarian cancer syndrome according to National Comprehensive Cancer Network eligibility criteria, their lifetime breast cancer risk was greater than 20% according to the Claus model, or their 5-year breast cancer risk was greater than 1.7% according to the CARE model (African American women) or the modified Gail model (all other women). Women who did not meet any of these criteria were classified as average risk. The software tool generates the result from each assessment instrument along with clinical decision support that includes recommendations for chemoprevention, enhanced screening with breast magnetic resonance imaging, and genetic counseling referral based on the result of the assessments and national practice guidelines. Primary care clinicians received a hard copy of BCRA results along with clinical decision support at the time of the participant encounter, and participants received the result of BCRA directly from their primary care clinician. The study protocol did not include any decision aids or educational materials for study participants. Patient education was nonstandardized and was left to the discretion of each treating clinician.

The primary outcome was performance of a mammogram. Participants’ medical records were reviewed from August 5, 2016, to March 5, 2017, to abstract mammography data. A survey administered at the time of study enrollment (before participants learned their risk status) collected demographic information and measures of breast cancer beliefs. The survey instrument is described in eTable 2 in the Supplement. The enrollment survey was translated into Spanish and was administered in either English or Spanish by bilingual research staff according to participants’ preference. Both the English version and the Spanish translation of the enrollment survey were pretested with cognitive interviewing with a purposive sample of individuals with a family history of breast cancer who did not participate in the main study to ensure comprehension and identify any question or response problems in the survey instrument.

### Statistical Analysis

Characteristics of high-risk participants were compared with those of average-risk participants using logistic regression models weighted to account for unequal probability of recruitment. Generalized estimating equations were used to compare the rate of mammography during 18 months of usual care before BCRA with the rate during 18 months following BCRA and assess factors associated with mammography use. This approach accounts for undersampling of average-risk participants and for correlation between pre- and post-BCRA data. Unadjusted bivariate analyses with generalized estimating equations were performed by combining all events (performance of a screening mammogram) occurring during the 18 months of usual care before BCRA with all events occurring over 18 months after participants underwent BCRA. A multivariable model was estimated that included factors associated with mammography at a level of *P* < .20 in unadjusted models. Generalized estimating equation models specified equal within-group correlations (exchangeable correlation structure) among repeated measures. The prespecified analysis plan included subgroup analyses stratified by risk level. All *P* values were 2-sided and considered significant at *P* < .05. Analyses were performed with Stata, version 16 (StataCorp LLC).

## Results

A total of 267 women at average risk and 224 women at high risk of breast cancer were invited to participate in the prospective cohort study, and 186 women (69.7%) with an average risk and 161 women (71.9%) with high risk completed study enrollment. This analysis included 98 (52.7%) average-risk and 90 (55.9%) high-risk participants who were older than 40 years at the time of study enrollment (eFigure in the Supplement). Mean [SD] age of participants included in this analysis was 50.8 [7.04] years; 70 women (37.2%) self-identified as Hispanic, 114 (60.6%) as non-Hispanic African American, and 4 (2.1%) as other racial and ethnic groups (4 non-Hispanic White women); for the purposes of the analysis we combined all non–African American participants into a single group. Demographic characteristics and baseline beliefs for this socioeconomically disadvantaged cohort are reported in Table 1. Compared with women at average risk, women at high risk were more likely to be non-Hispanic African American (62 [68.9%] vs 52 [53.1%]), more likely to report that they discussed their breast cancer risk with their primary care clinician (20 [22.2%] vs 12 [12.2%]), less likely to correctly identify their level of breast cancer risk before learning the result of their BCRA (41 [45.6%] vs 70 [71.4%]), and more likely to report a moderate or high level (41 [45.6%] vs 33 [33.7%]) of breast cancer worry.

**Table 1.** Characteristics of Study Participants^a^

Characteristic	No. (%)			*P* value
	All participants	Risk		
		High	Average	
No. of participants	188	90	98	
Race and ethnicity				
Hispanic	70 (37.2)	25 (27.8)	45 (45.9)	.03
Non-Hispanic African American	114 (60.6)	62 (68.9)	52 (53.1)	
Other^b^	4 (2.1)	3 (3.3)	1 (1.0)	
Birth place				
US	117 (62.2)	64 (71.1)	53 (54.1)	.01
Other	63 (33.5)	22 (24.4)	41 (41.8)	
No response	8 (4.3)	4 (4.4)	4 (4.1)	
Age, y				
40-49	87 (46.3)	35 (38.9)	52 (53.1)	.05
50-69	101 (53.7)	55 (61.1)	46 (46.9)	
Marital status				
Married/partner	85 (45.2)	38 (42.2)	47 (48.0)	.44
Single/no partner	97 (51.6)	49 (54.4)	48 (49.0)	
No response	6 (3.2)	3 (3.3)	3 (3.0)	
Educational level				
≤High school	99 (52.7)	36 (40.0)	63 (64.3)	.001
>High school	84 (44.7)	51 (56.7)	33 (33.7)	
No response	5 (2.7)	3 (3.3)	2 (2.0)	
Employment status				
Employed	92 (48.9)	45 (50.0)	47 (48.0)	.76
Unemployed	88 (46.8)	41 (45.6)	47 (48.0)	
No response	8 (4.3)	4 (4.4)	4 (4.0)	
Annual household income, $				
≤20 000	117 (62.2)	51 (56.7)	66 (67.3)	.07
>20 000	60 (31.9)	35 (38.9)	25 (25.5)	
No response	11 (5.9)	4 (4.4)	7 (7.1)	
Health insurance				
Medicaid/Medicare	166 (88.3)	77 (85.6)	89 (90.8)	.27
Private	22 (11.7)	13 (14.4)	9 (9.2)	
Clinician ever talked about BC risk				
No	114 (60.6)	44 (48.9)	70 (71.4)	.02
Yes	32 (17.0)	20 (22.2)	12 (12.2)	
Unsure	42 (22.3)	26 (28.9)	16 (16.3)	
Perceived health status				
Excellent/good	135 (71.8)	61 (67.8)	74 (75.5)	.23
Poor/fair	47 (25.0)	26 (28.9)	21 (21.4)	
No response	6 (3.2)	3 (3.3)	3 (3.1)	
Perceived BC susceptibility^c^				
Not increased	103 (54.8)	33 (36.7)	70 (71.4)	<.001
Increased	63 (33.5)	41 (45.6)	22 (22.5)	
No response	22 (11.7)	16 (17.8)	6 (6.1)	
BC cultural beliefs, mean (SD)^c^^,^^d^	2.04 (2.20)	2.05 (2.01)	2.04 (2.34)	.97
Cancer fatalism, mean (SD)^c^^,^^e^	1.86 (2.14)	2.27 (2.16)	1.53 (2.08)	.07
BC worry^c^				
Low	99 (52.7)	38 (42.21)	61 (62.2)	.004^f^
Moderate	46 (24.5)	24 (26.7)	22 (22.4)	
High	28 (14.9)	17 (18.9)	11 (11.2)	
No response	15 (7.9)	11 (12.2)	4 (4.1)	

Abbreviation: BC, breast cancer.

^a^ Characteristics of participants at high risk were compared with those at average risk using bivariate, weighted logistic regression.

^b^ Other race and ethnicity included 4 non-Hispanic White women.

^c^ Description of scales used to measure perceived BC risk, BC cultural beliefs, fatalism, and BC worry is presented in eTable 2 in the Supplement. Categorized as an ordinal variable for weighted logistic regression models.

^d^ Score range is 0 to 15; higher score indicates more cultural beliefs that may pose barriers to obtaining a mammogram.

^e^ Score range is 0 to 11; higher score indicates more fatalistic views regarding development and outcome of breast cancer.

^f^ *P* value is for BC worry as a continuous variable on scale of 0 to 4.

There was a nonsignificant increase in the overall rate of mammography use, from 38.8% during usual care to 48.9% after BCRA (*P* = .12) (Figure). The rate of mammography significantly increased among women at high risk (36.6% during usual care and 51.1% following BCRA; *P* = .02). There was a nonsignificant numeric increase among average-risk participants (40.8% during usual care and 46.9% after BCRA; *P* = .30).

**Figure.**
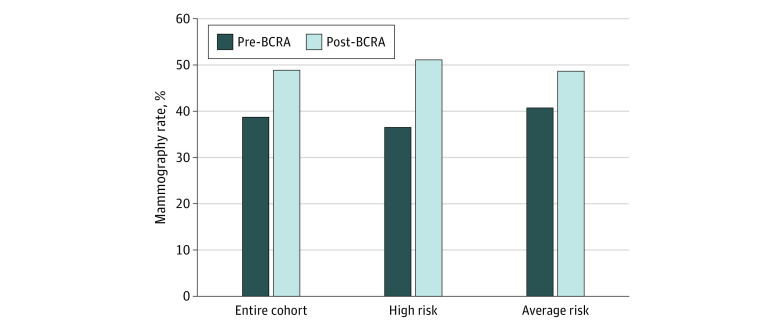
Change in Mammography Use Following Breast Cancer Risk Assessment BCRA indicates breast cancer risk assessment.

Table 2 displays variables associated with mammography use in unadjusted analyses. A high level of breast cancer worry was associated with mammography use in bivariate analysis (odds ratio [OR], 2.31; 95% CI, 1.00-5.34), but the level was not significant in multivariable models (OR, 1.80; 95% CI, 0.70-4.63) (Table 3). Performance of BCRA was significantly associated with mammography use among high-risk participants in unadjusted models (OR, 1.81; 95% CI, 1.09-2.99), but the association was not significant for the entire cohort (OR, 1.37; 95% CI, 0.92-2.03) or for the average-risk subgroup (OR, 1.28; 95% CI, 0.80-2.06). In multivariable analysis (Table 3), performance of BCRA remained associated with mammography use among women at high risk (OR, 1.88; 95% CI, 1.10-3.23). No other variables were significant in multivariable models. Eleven participants in the high-risk subgroup attended a genetic counseling session after BCRA. A sensitivity analysis that excluded the 11 high-risk participants who attended a genetic counseling session in the multivariable model showed a modest attenuation of the association between BCRA and mammography use (OR, 1.65; 95% CI, 0.92-2.95).

**Table 2.** Factors Associated With Mammography Use in Unadjusted Analyses

Variable	Participants, OR (95% CI)^a^		
	All	Risk	
		High	Average
No. of participants	188	90	98
Time			
Usual care	1 [Reference]	1 [Reference]	1 [Reference]
After BCRA	1.37 (0.92-2.03)	1.81 (1.09-2.99)^b^^,^^c^	1.28 (0.80-2.06)
Race and ethnicity			
Non–African American^d^	1 [Reference]	1 [Reference]	1 [Reference]
African American	0.68 (0.39-1.17)	0.96 (0.47-1.95)	0.63 (0.33-1.19)
Age, y			
40-49	1 [Reference]	1 [Reference]	1 [Reference]
50-69	1.25 (0.73-2.14)	1.92 (0.96-3.82)	1.15 (0.60-2.18)
Birthplace			
Outside the US	1 [Reference]	1 [Reference]	1 [Reference]
US	0.63 (0.36-1.12)	0.80 (0.38-1.72)	0.60 (0.31-1.15)
Marital status			
Single/no partner	1 [Reference]	1 [Reference]	1 [Reference]
Married/partner	1.11 (0.64-1.93)	1.85 (0.95-3.62)	0.99 (0.52-1.91)
Educational level			
>High school	1 [Reference]	1 [Reference]	1 [Reference]
≤High school	1.11 (0.64-1.92)	1.13 (0.58-2.22)	1.12 (0.56-2.23)
Employment status			
Employed	1 [Reference]	1 [Reference]	1 [Reference]
Unemployed	1.35 (0.78-2.34)	0.94 (0.48-1.83)	1.47 (0.77-2.83)
Annual household income, $			
>20 000	1 [Reference]	1 [Reference]	1 [Reference]
≤20 000	0.89 (0.50-1.58)	0.92 (0.47-1.81)	0.89 (0.42-1.87)
Health insurance			
Private	1 [Reference]	1 [Reference]	1 [Reference]
Medicaid/Medicare	0.73 (0.28-1.91)	0.63 (0.25-1.59)	0.76 (0.25-2.30)
Clinician ever talked about BC risk			
No	1 [Reference]	1 [Reference]	1 [Reference]
Yes	1.78 (0.89-3.53)	1.51 (0.65-3.47)	1.98 (0.73-5.38)
Perceived health status			
Excellent/good	1 [Reference]	1 [Reference]	1 [Reference]
Poor/fair	1.13 (0.61-2.12)	0.93 (0.45-1.93)	1.19 (0.54-2.63)
Perceived BC susceptibility			
Not increased	1 [Reference]	1 [Reference]	1 [Reference]
Increased	1.10 (0.61-1.97)	0.96 (0.46-1.99)	1.18 (0.55-2.56)
BC cultural beliefs^b^^,^^e^	0.99 (0.88-1.12)	0.88 (0.70-1.09)	1.01 (0.86-1.17)
Cancer fatalism^b^^,^^e^	1.09 (0.95-1.25)	0.91 (0.75-1.10)	1.15 (0.95-1.38)
BC worry^b^^,^^e^			
Low	1 [Reference]	1 [Reference]	1 [Reference]
Moderate	0.81 (0.42-1.54)	0.98 (0.47-2.05)	0.77 (0.35-1.70)
High	2.31 (1.00-5.34)^f^	1.38 (0.51-3.69)	2.88 (0.93-8.94)

Abbreviations: BC, breast cancer; BCRA, breast cancer risk assessment; OR, odds ratio.

^a^ Unadjusted bivariate analyses with generalized estimating equations were performed by combining all events (performance of a screening mammogram) occurring during the 18 months of usual care before BCRA with all events occurring over 18 months after participants underwent BCRA.

^b^ Categorized as an ordinal variable for generalized estimating equations.

^c^ Significant at *P* = .02.

^d^ The non-African American category included 70 Hispanic women, and 4 non-Hispanic White women.

^e^ Measures described in eTable 2 in the Supplement.

^f^ Significant at *P* = .05.

**Table 3.** Multivariable Analysis of Factors Associated With Mammography Use^a^

Variable	Participants, OR (95% CI)	
	All	High risk
Time		
Usual care	1 [Reference]	1 [Reference]
After BCRA	1.30 (0.81-2.09)	1.88 (1.10-3.23)^b^
Race and ethnicity		
Non–African American^c^	1 [Reference]	NA^d^
African American	1.60 (0.44-5.92)	NA^d^
Age, y		
40-49	NA^d^	1 [Reference]
50-69	NA^d^	1.78 (0.86-3.64)
Birthplace		
Outside the US	1 [Reference]	NA^d^
US	0.43 (0.12-1.54)	NA^d^
Marital status		
Single/no partner	NA^d^	1 [Reference]
Married/partner	NA^d^	1.94 (0.97-3.88)
Clinician ever talked about BC risk		
No	1 [Reference]	NA^d^
Yes	1.51 (0.74-3.10)	NA^d^
BC worry		NA^d^
Low	1 [Reference]	NA^d^
High	1.80 (0.70-4.63)	NA^d^

Abbreviations: BC, breast cancer; BCRA, breast cancer risk assessment; NA, not applicable; OR, odds ratio.

^a^ Multivariable analysis with generalized estimating equations was conducted by combining all events (performance of a screening mammogram) occurring during the 18 months of usual care before BCRA with all events occurring during 18 months after participants underwent BCRA.

^b^ Significant at *P* = .02.

^c^ The non-African American category included 70 Hispanic women, and 4 non-Hispanic White women.

^d^ Covariates with *P* > .2 in unadjusted analyses were not included in multivariable models.

## Discussion

This prospective cohort study found that incorporating BCRA into routine preventive health care was associated with improved use of screening mammography among women of underserved racial and ethnic minority groups who had a high risk of breast cancer. A previous report described the feasibility of a technology-enabled approach to population screening for breast cancer risk^22^; 100% of 1269 women who were eligible for BCRA completed the assessment while they waited to see their primary care clinician. This strategy for conducting risk assessment involved minimal change to clinic workflow overall, requiring only 2 to 3 extra minutes of staff time on average to input family history and nonfamilial risk factor data and generate the assessment report.^22^ The process did not require any changes in physician workflow, nor did it require scheduling longer appointment slots for annual well visits or new-patient visits.

Less than half of all women at high risk (45.6%) correctly perceived their risk status, compared with more than 70% of women at average risk (71.4%). This difference is consistent with other reports in the literature suggesting that approximately half of African American women accurately perceive their breast cancer risk.^27,28^ Somewhat unexpectedly, perceived risk was not associated with mammography use in the entire cohort or in either of the risk-stratified subgroups. Kim and colleagues^27^ reported similar findings for a racially diverse cohort from San Francisco. Qualitative research with African American women with hereditary breast cancer risk found that decisions to engage in cancer risk management behaviors are associated with 3 accumulated layers of risk information: perceived risk at the foundational layer, general information about managing breast cancer risk at the middle layer, and specific information about risk-management options at the most proximal layer.^29^ African American women experience distinct dynamics at each of these layers compared with non-Hispanic White women. The authors noted that information access is associated with clinician access.^29^ Consistent with that finding, we observed a numeric increase in mammography adherence among women who ever discussed breast cancer risk with a clinician in the entire cohort and in both risk-level subgroups, although the increase did not reach statistical significance. These data suggest that coupling individualized risk estimates with tailored educational material and decision aids may stimulate discussion with clinicians and may be more useful in motivating engagement in breast cancer screening than simply delivering risk estimates, as done in this study.

One potential unintended consequence of widespread implementation of BCRA in asymptomatic women is the possibility that the information would provide a false sense of security for women at average risk and reduce mammography use in that subgroup; however, we did not observe a signal of that adverse outcome. Although the study was underpowered for the average-risk subgroup, we observed a nonsignificant numeric increase in mammography uptake in that group as well as in the high-risk group. This increase suggests that providing individualized risk estimates might be associated with improved mammography use in women of underserved racial and ethnic minority groups regardless of risk status, although that hypothesis will need to be tested in an adequately powered study.

A large body of research reports strategies for improving mammography adherence in women of racial and ethnic minority groups, including patient reminders and educational/motivational interventions, and patient navigation.^30^ The present study did not incorporate any interventions designed specifically to facilitate mammography adherence (eg, vouchers, reminders, patient navigation to assist in scheduling appointments or address other barriers). Despite this lack of interventions, the improvement in mammography adherence rates associated with BCRA in our study compares favorably with interventions designed specifically for that purpose.^30^ A multilevel approach that identifies the high-risk population with BCRA in primary care settings would allow other interventions that improve adherence to mammography (eg, patient navigation) to be targeted to the subgroup of women who benefit the most from screening. A strategy that combines BCRA with navigation of high-risk individuals may be more useful than either intervention alone for addressing breast cancer disparities, and this approach is supported by a cost-effectiveness analysis.^31^ In addition, this multilevel approach could be tested as a strategy to increase use of enhanced cancer control measures, such as genetic counseling and breast magnetic resonance imaging screening. More research is needed to assess whether this strategy would mitigate racial disparities in breast cancer mortality.

The US Preventive Services Task Force recently noted that the benefits of screening for hereditary breast cancer risk have not been directly evaluated by research.^15^ To our knowledge, this is the first study to observe improved uptake of a clinical procedure (ie, screening mammogram) following systematic BCRA in women without a personal history of breast cancer. The present study was not designed to detect change in a clinical outcome (eg, breast cancer incidence or mortality). More work is needed with studies that have longer follow-up and are adequately powered to detect changes in relevant clinical outcomes. However, this study provides data supporting the feasibility of research designed to assess the clinical outcomes associated with BCRA in women of underserved racial and ethnic minority groups.

### Limitations

This study has limitations. The investigation was conducted in urban FQHCs with African American and Hispanic women. The results need to be confirmed in other settings and with women from other racial and ethnic groups. In addition, all women in this study had either public or private health insurance. The result may have been different in a population that includes women without health insurance. We did not measure health literacy or numeracy and we did not provide standardized educational material or decision aids, which could have confounded the results. It is unknown whether the benefit will be maintained over the long term and whether similar improvements would be seen in women with higher baseline screening rates. This study did not examine whether the association between BCRA and mammography use in women at high risk was owing to a change in primary care clinician recommendations or patient adherence. Differences in baseline characteristics between the high-risk and average-risk subgroups could contribute to the observed differential association with BCRA. The cohort design prevented us from drawing conclusions on causality, and the study was underpowered to detect differences in the average-risk subgroup.

## Conclusions

This study suggests that an individualized approach to breast cancer screening enabled by implementation of BCRA in FQHCs is associated with increased use of screening mammography among women of medically underserved racial and ethnic minority groups who have an increased risk of developing breast cancer. This approach warrants further study as a strategy to reduce racial disparities in breast cancer mortality, although further research should combine BCRA with other interventions that may improve mammography adherence to maximize the benefit.

## Notes

Supplement. **eTable 1.** Risk Assessment Instruments **eTable 2.** Survey Instruments **eFigure.** CONSORT Diagram
eReferences
