# Supplementary material for: Association of Population Screening for Breast Cancer Risk With Use of Mammography Among Women in Medically Underserved Racial and Ethnic Minority Groups
Source: JAMA Netw Open. 2021 Sep 10;4(9):e2123751. doi: 10.1001/jamanetworkopen.2021.23751 (PMC8433603; doi:10.1001/jamanetworkopen.2021.23751)
Supplement: Supplement. — eTable 1. Risk Assessment Instruments eTable 2. Survey Instruments eFigure. CONSORT Diagram eReferences [file jamanetwopen-e2123751-s001.pdf]

Supplementary Online Content

Schwartz C, Chukwudozie IB, Tejeda S, et al. Association of population screening for breast cancer risk with use of mammography among women in medically underserved racial and ethnic minority groups. *JAMA Netw Open*. 2021;4(9):e2123751. doi:10.1001/jamanetworkopen.2021.23751

eTable 1. Risk Assessment Instruments

eTable 2. Survey Instruments

eFigure. CONSORT Diagram

eReferences

This supplementary material has been provided by the authors to give readers additional information about their work.

© 2021 Schwartz C et al. *JAMA Network Open.*

eTable 1. Risk Assessment Instruments

Instrument	Purpose For This Study	Risk Factors Included	Comments
Modified Gail model[1]	Provide 5-yr risk estimates for non-African American women to identify candidates for chemoprevention[2]	Age, age menarche and first live birth, number prior breast biopsies, presence of atypia, number FDR w/ breast cancer	Provides 5-yr and lifetime breast cancer risk estimates. Available as the NCI Breast Cancer Risk Assessment Tool. FDA indication for chemoprevention with tamoxifen or raloxifene to reduce breast cancer risk is based on 5-yr Gail model estimate.
CARE model[3]	Provide 5-yr risk estimates for African American women to identify candidates for chemoprevention[2]	Same as Modified Gail model	Developed for African Americans. Available as the NCI Breast Cancer Risk Assessment Tool in combination with the modified Gail model.
Claus model[4]	Identify candidates for breast MRI screening[5] and provide lifetime risk estimate based on family history	Age, number FDR and SDR w/ breast cancer and their age at diagnosis	Provides cumulative lifetime breast cancer risk estimates up to age 79 for several family history scenarios. American Cancer Society endorses this model for identifying candidates for breast MRI screening as adjunct to mammography[5]
Pedigree Assessment Tool (PAT)[6,7]	Identify candidates for genetic counseling referral due to high risk for hereditary breast cancer syndrome	Number of FDR, SDR and TDR with female or male breast cancer, ovarian cancer, and bilateral breast cancer; ages at diagnosis, and Ashkenazi Jewish ancestry.	Weighted point scoring system with points assigned for each cancer diagnosis in both parental lineages. Final score indicates whether genetic counseling referral is indicated (PAT score $\geq$ 8) or not (PAT score < 8). The PAT was endorsed by the USPSTF as a screening tool in primary care to identify candidates for genetic counseling referral.[8] For this study the PAT was supplemented with NCCN® criteria, version 1.2011[9] to identify candidates who met NCCN criteria for genetic testing but had PAT score < 8. Those women were recommended for Genetic Counseling referral in addition to women with PAT score $\geq$ 8.

Abbreviations: FDR, first-degree relative; SDR, second-degree relative; TDR, third-degree relative;

eTable 1. A custom software application was created for this study that collected the necessary breast cancer risk information necessary to perform an assessment with each model in the table. An integrated report that included the results from each model and clinical decision support with individualized recommendations for screening, prevention and genetic counseling referral based on results of the assessments were provided to the primary care physician at the time of the patient encounter. Source code for the Modified Gail Model and Care Model were provided by M. Gail and D. Pee. For the other models, code was written for this project by Godar Custom Software, Inc.

© 2021 Schwartz C et al. *JAMA Network Open.*

eTable 2. Survey Instruments

Scale	Number of Items	Scoring
Perceived Breast Cancer Susceptibility[10]	1	5-point Likert scale to question: "Your chances of getting breast cancer are higher than most women your age." Strongly agree/agree coded as perceived susceptibility increased; neutral/disagree/strongly disagree coded as perceived susceptibility not increased.
Breast Cancer Cultural Beliefs[11]	15	True/False, total score is sum of all responses, with range 0-15. Higher score= more cultural beliefs that could pose a barrier to obtaining a mammogram.
Breast Cancer Worry[12]	1	5-point Likert scale to question: "How worried are you about getting breast cancer someday?" higher score= more worry. For the analysis responses were collapsed into 3 categories; low (0-1), medium (2) and high worry (3-4)
Cancer Fatalism[13]	11	Yes/No, total score is sum of all responses, with range 0-11. Higher score= more fatalistic view of cancer development and outcome

© 2021 Schwartz C et al. *JAMA Network Open.*

eFigure. CONSORT Diagram

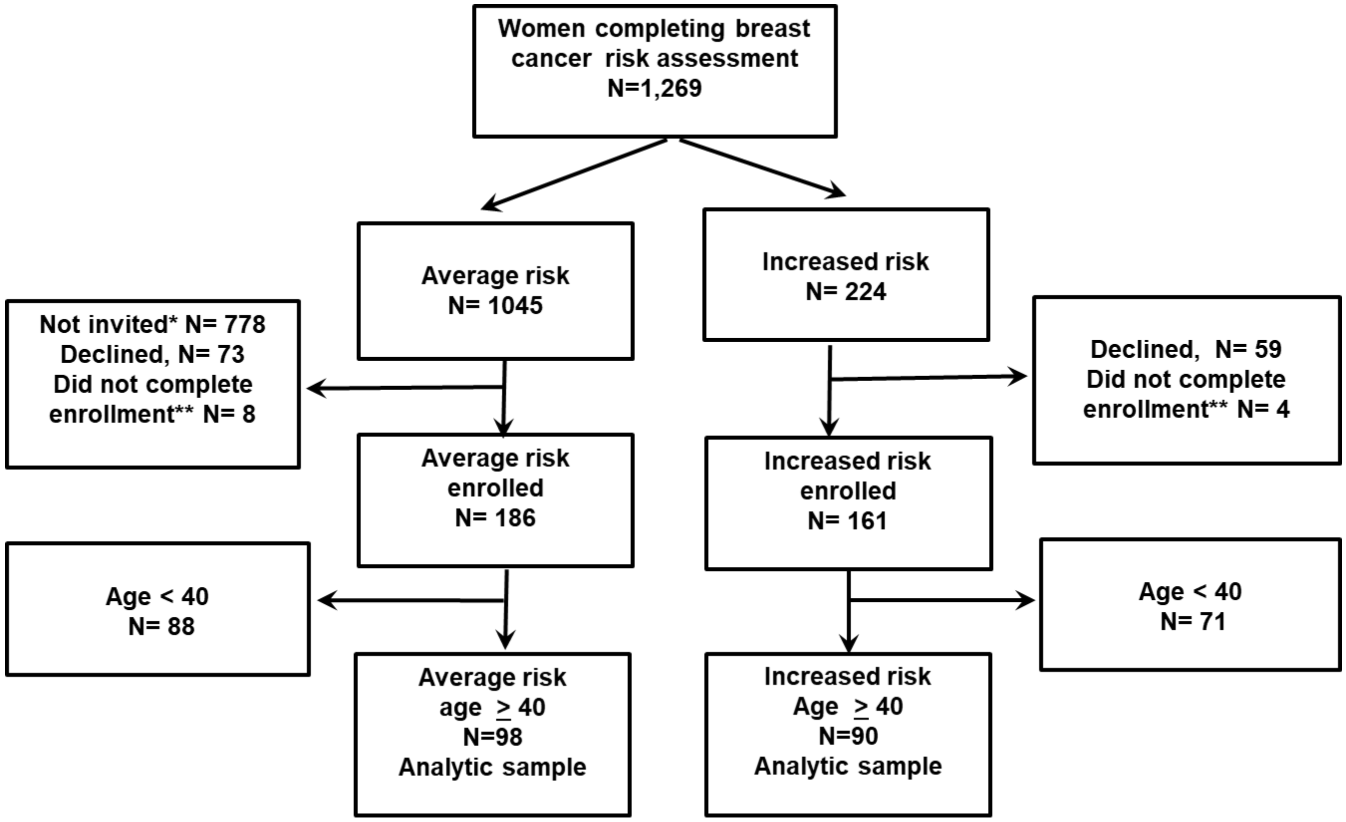

*The study was designed to randomly invite 1 out of 4 average risk women to participate in the study.

**Women who did not complete all enrollment activities before the encounter with the provider were excluded.

© 2021 Schwartz C et al. *JAMA Network Open.*

eReferences

1.   Costantino JP, Gail MH, Pee D, Anderson S, Redmond CK, Benichou J, Wieand HS. Validation studies for models projecting the risk of invasive and total breast cancer incidence. J Natl Cancer Inst. 1999 Sep 15;91(18):1541-8.

2. National Comprehensive Cancer Network Clinical Practice Guidelines in Oncology. Breast Cancer Risk Reduction, version 1.2020. Accessed at https://www.nccn.org/professionals/physician_gls/pdf/breast_risk.pdf on January 14, 2021.

3. Gail MH, Costantino JP, Pee D, Bondy M, Newman L, Selvan M, Anderson GL, Malone KE, Marchbanks PA, McCaskill-Stevens W, Norman SA, Simon MS, Spirtas R, Ursin G, Bernstein L. Projecting individualized absolute invasive breast cancer risk in African American women. J Natl Cancer Inst. 2007 Dec 5;99(23): 1782-92.

4. Claus EB, Risch N, Thompson WD. Autosomal dominant inheritance of early-onset breast cancer. Implications for risk prediction. Cancer. 1994;73: 643-651.

5. Saslow D, Boetes C, Burke W, et al. American Cancer Society guidelines for breast screening with MRI as an adjunct to mammography. CA Cancer J Clin. 2007;57: 75-89.

6. Hoskins KF, Zwaagstra A, Ranz M. Validation of a Tool for Identifying Women at High Risk for Hereditary Breast Cancer in Population-Based Screening. *Cancer* 2006;107: 1769-1776.

7. Teller P, Hoskins KF, Zwaagstra A, Iyengar R, Rizzo M, Stanislaw C, Gabram S. Validation of the Pedigree Assessment Tool in Families with BRCA1 & BRCA2 Mutations. *Annals of Surgical Oncology.* 2010; 17(1): 240-247.

8. Moyer VA; U.S. Preventive Services Task Force. Risk assessment, genetic counseling, and genetic testing for BRCA-related cancer in women: U.S. Preventive Services Task Force recommendation statement. Ann Intern Med. 2014 Feb 18;160(4):271-81.

9. National Comprehensive Cancer Network Clinical Practice Guidelines: Genetic/Familial High Risk Assessment: Breast and Ovarian, version 1.2011. Current version available at https://www.nccn.org/professionals/physician_gls/pdf/genetics_bop.pdf

10. Champion VL, Monahan PO, Springston JK, Russell K, Zollinger TW, Saywell RM Jr, Maraj M. J Health Psychol. 2008 Sep;13(6):827-37.

11. Ferrans C, Rauscher G, Akpan B, et al. Cultural beliefs contributing to disparities in later-stage breast cancer among newly diagnosed African American, Latina, and Caucasian women. Oncol Nurs Forum. 2007; 34:180–1.

12. Lerman C, Trock B, Rimer BK, Jepson C, Brody D, Boyce A. Health Psychol. 1991;10(4):259-67.

13. Mayo, R. M., Ureda, J. R., Parker, V. G. Importance of fatalism in understanding mammography screening in rural elderly women. Journal of Women and Aging. 2001;13(1): 57-72.

© 2021 Schwartz C et al. *JAMA Network Open.*
